# An Unusual Presentation with Facial Hyperpigmentation on Escalation of the Dose of Sertraline

**DOI:** 10.1155/2024/7416277

**Published:** 2024-08-07

**Authors:** Omkar Dhungel, Indra Prasad Amatya, Pawan Sharma

**Affiliations:** Institute of Medicine Patan Academy of Health Sciences, Lalitpur, Nepal

## Abstract

**Background:**

Hyperpigmentation is a common side effect of different drugs with many of these having a well-explained mechanism and some even having a characteristic distribution. However, it is a rare side effect of sertraline, a selective serotonin reuptake inhibitor (SSRI), with only a few reported cases. In addition, there are no specific characteristics of the lesions or the risk factors. *Case Summary*. This is a case report of a 24-year-old male with panic disorder, who developed hyperpigmentation over the face after 5 days of increasing the dosage of sertraline to 100 mg/day. There were no other significant findings from the physical examination or investigations. The patient was treated as a case of sertraline-induced hyperpigmentation, and the dose was reduced to 75 mg/day and maintained at 50 mg/day after 1 week along with tablet propranolol 20 mg/day. He was also prescribed tablet tranexamic acid 500 mg/day and sunscreen with sun protection factor 50. The hyperpigmentation disappeared within 2 months, and the medication was gradually tapered after 7 months of treatment.

**Conclusion:**

Hyperpigmentation is a rare but distressing side effect of sertraline. It is a potentially curable side effect if recognized early. Early recognition and intervention can decrease unnecessary investigations and treatment. There are limited studies highlighting this unusual adverse effect of this commonly used SSRI. Hence, further studies are needed to better understand various aspects of this condition including the characteristics, patients at risk, and possible management. The development of diagnostic and treatment guidelines would decrease the dilemma of identification and management.

## 1. Introduction

Sertraline is a selective serotonin reuptake inhibitor (SSRI) that allosterically inhibits the serotonin transporter (SERT), thereby increasing the concentration of serotonin (5-HT) in the synapse [1]. SSRIs like sertraline are one of the most commonly used drugs for various psychiatric conditions. Sertraline is approved by the US Food and Drug Administration (FDA) for major depressive disorder (MDD), obsessive–compulsive disorder (OCD), posttraumatic stress disorder (PTSD), premenstrual dysphoric disorder (PMDD), panic disorder, and social anxiety disorder (SAD) [2]. Common side effects of sertraline include weight gain, drowsiness, insomnia, reduced libido, increased ejaculation latency, serotonin syndrome, and hyponatremia caused by syndrome of inappropriate antidiuretic hormone (ADH) secretion (SIADH) [1]. Although a few publications have reported cases with various forms of skin complications, such as erythema multiforme, Stevens–Johnson syndrome, drug-induced hypersensitivity syndrome, alopecia, hypertrichosis, leukocytoclastic vasculitis, and acneiform eruption, all cutaneous side effects of antidepressants demand assessment [3, 4]. Hyperpigmentation following the use of sertraline has been reported in some cases [5, 6], but it is interesting to note that there is no specific distribution, time of onset, or characteristic among them. Hence, it is important to know about this rare side effect of sertraline. This is a case of a 24-year-old male with panic disorder, who was treated with sertraline 25 mg/day for 4 days, which was increased gradually to 75 mg/day over the next 5 weeks. As the symptoms persisted, the dose was increased to 100 mg/day, and he developed hyperpigmentation over the face after 5 days.

## 2. Case Presentation

**Figure 1:**
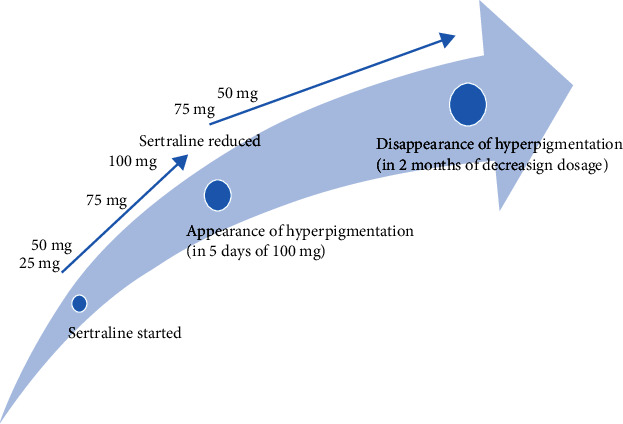
Timeline of sertraline-induced hyperpigmentation.

The patient was a 24-year-old unmarried, day laborer with no history of diagnosed psychiatric illness in his family or himself. He complained of episodes of spontaneous-onset fearfulness, palpitations, shortness of breath, choking sensation, and fear of impending death for the last 4 years. It would persist for about 10–20 min at a time, with spontaneous recovery, and would occur two to five times a week. He had not sought consultation prior. As the frequency of symptoms increased and hampered his daily life for 1 month, he attended a psychiatric consultation. He was diagnosed according to the International Classification of Diseases, 11th Revision (ICD-11), as a case of panic disorder (6B01) and treated with tablet sertraline 25 mg/day for 4 days and then 50 mg/day for 3 weeks. As there was a partial improvement in anxiety symptoms, the dosage of sertraline was increased to 75 mg/day in another 2 weeks. There was a slight improvement in panic attacks, so the dose was increased to 100 mg per day. After 5 days of optimizing sertraline to 100 mg/day, he came for a follow-up with the complaint of blackish discoloration over his face (Figure 1). His baseline blood investigations including thyroid function test and echocardiogram were normal. There was no history of allergy to food or drugs and no history of recent fever, use of other medications, migration, change in work, or excess sun exposure.

**Figure 2:**
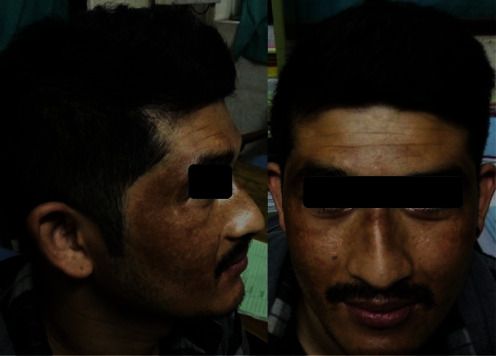
Five days after treatment with sertraline 100 mg daily.

On examination, there was hyperpigmentation involving the malar region, temple, and nasal ridge (Figure 2). There was no pruritus, macule, papule, discharge, erosion, or ulcer, and the hyperpigmentation was not present in other parts of the body. The hyperpigmentation gradually increased in size and color intensity, so he came for an early follow-up. His vitals were within normal limits, and there were no other abnormalities in the general physical and systemic examinations.

**Figure 3:**
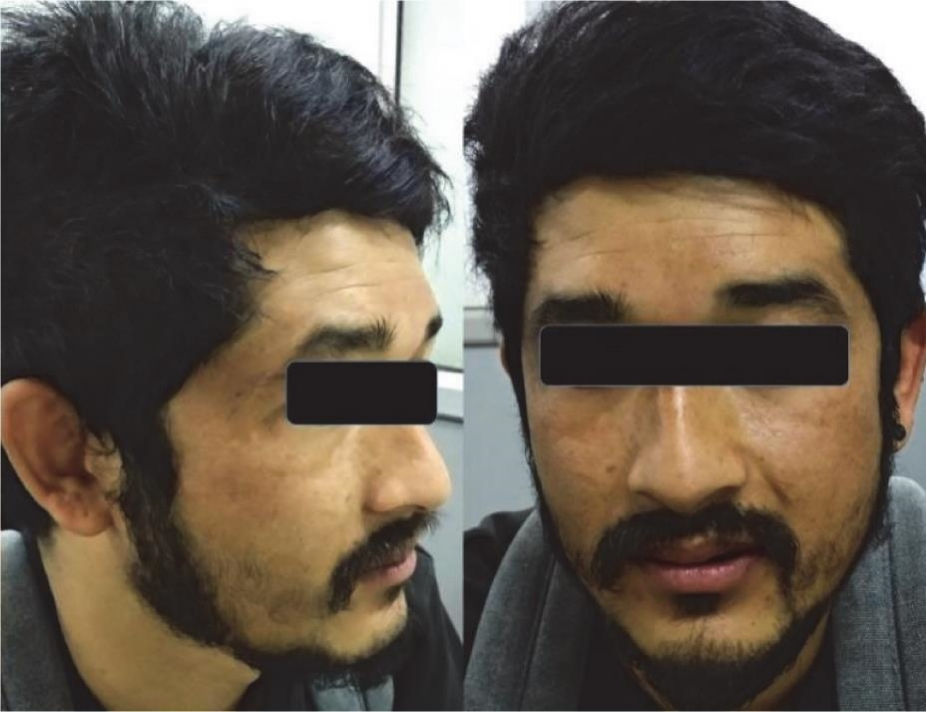
Two months after decreasing sertraline to 50 mg daily.

The possibilities of SSRI-induced hyperpigmentation were discussed among psychiatrists, and the patient was referred to dermatology and rheumatology specialists to rule out other possible causes. As there was an improvement in panic attacks, the consensus was made to continue sertraline in a decreased dose. Sertraline was tapered to 75 mg/day and maintained at 50 mg/day after 1 week, with the addition of tablet propranolol 20 mg/day for hand tremors. There was no aggravation of anxiety symptoms. The dermatologist treated him with tablet tranexamic acid 500 mg per day and sunscreen with sun protection factor 50, with differential diagnoses of sertraline-induced hyperpigmentation or melasma. There is an off-label use of tranexamic acid (250–1500 mg per day) in melasma and different types of hyperpigmentation [7]. The patient was treated for 1 month by the dermatologist. The rheumatological evaluation was normal and needed no intervention. In the 2-month follow-up, the hyperpigmentation had decreased significantly (Figure 3). The patient maintained well on sertraline 50 mg/day and propranolol 20 mg/day for 7 months. He was followed up bimonthly. When the patient had been symptom-free of anxiety for 6 months, the medications were gradually tapered off over the next 2 months. He was advised to follow-up if any such symptoms appear in the mildest form or disturbance in sleep or appetite and advised to continue deep breathing exercise as advised earlier.

## 3. Discussion

This case report highlights a rare side effect of sertraline, i.e., facial hyperpigmentation. Although the exact mechanism of hyperpigmentation caused by sertraline and other antidepressants is unknown, alpha-melanocyte-stimulating hormone (a-MSH) which stimulates tyrosinase and converts tyrosine to melanin may play a role [6]. Since dopamine, norepinephrine, and serotonin are by-products of amino acids like tyrosine and tryptophan, their involvement may point to the possible mechanism of hyperpigmentation [2]. A review article mentioned that certain characteristics such as female gender, increasing age, African American ethnicity, use of multiple medications, and presence of a serious illness place an individual at higher risk for adverse cutaneous drug reactions due to antidepressants [8]. On the contrary, the case presented here had no such mentioned characteristics. There are reported cases of melanosis with citalopram and clomipramine, whereas leukoderma has been associated with a case treated with venlafaxine [8]. Among the antidepressants, tricyclic antidepressants (TCAs) like imipramine and clomipramine have mostly been reported to cause hyperpigmentation. Various cutaneous effects of SSRIs were reviewed in May 2007 [4], but hyperpigmentation was not reported among any of the reviewed articles. A systematic review from 2013 found only one reported case of drug-induced hyperpigmentation caused by sertraline with evidence level 3 [9].

Hyperpigmentation is a rare but significant side effect of sertraline, which in some cases has been reported to persist even after discontinuation of the antidepressant [5]. In the case presented here, sertraline was not discontinued. There was a significant improvement in hyperpigmentation as the sertraline dosage was decreased and maintained at 50 mg/day.

There are some limitations to be considered. The patient was in parallel to a decreased sertraline dose, treated with tranexamic acid and sunscreen with protection factor 50, which may have contributed to the decreased hyperpigmentation observed. Also, a skin biopsy was not performed as the patient refused.

## 4. Conclusion

To conclude, sertraline is one of the most commonly used SSRIs and is effective in different psychiatric disorders. There are different well-documented side effects of sertraline, and hyperpigmentation is not on the list. This case report can potentiate the possible relationship between sertraline and hyperpigmentation. A clinician aware of this rare side effect can inform the patient prior and be vigilant during the treatment.

## Data Availability

Data sharing is not applicable to this article as no datasets were generated or analyzed during the current study.
